# First national survival data for colorectal cancer among Saudis between 1994 and 2004: what’s next?

**DOI:** 10.1186/1471-2458-13-73

**Published:** 2013-01-25

**Authors:** Mahmoud S Al-Ahwal, Yasmin H Shafik, Hazem M Al-Ahwal

**Affiliations:** 1 Department of Medicine, Colon Cancer Chair, Faculty of Medicine, King Abdulaziz University (KAU), Jeddah, Kingdom of Saudi Arabia; 2 Faculty of Medicine, King Abdulaziz University (KAU), Jeddah, Kingdom of Saudi Arabia; 3 Department of Surgery, Faculty of Medicine, King Abdulaziz University (KAU), Jeddah, Kingdom of Saudi Arabia

**Keywords:** Colorectal cancer, Saudi Arabia, Survival

## Abstract

**Background:**

Colorectal cancer (CRC) is the second most common malignancy in the Saudi population. This study aimed to review CRC data from the Saudi Cancer Registry (SCR) in order to evaluate the prognostic factors for CRC survival in Saudi patients.

**Methods:**

This study was a retrospective censored overall survival (OS) analysis of CRC data for the period 1994–2004 obtained from the SCR. Data were collected from all 13 administrative regions of the Kingdom of Saudi Arabia (KSA) by the SCR in collaboration with the National Information Center of the Ministry of Interior. The Kaplan-Meier method was used to calculate the cumulative survival rate, which was then stratified by gender and by period (1994–1999 versus 2000–2004). The clinico-pathological variables that might affect CRC survival were analyzed by Cox regression analysis.

**Results:**

Between 1994 and 2004, 549 CRC cases were diagnosed (363 [66.1%] in males and 186 [33.9%] in females). The OS for CRC during this period was 44.6% (44.7% for 1994–1999 and 44.3% for 2000–2004 [p=0.7]). There was a significant (p=0.003) discrepancy of 9.6% between the male five-year OS (41.0%) and the female five-year OS (50.6%). The five-year OS was 63.3% for patients with localized disease, 50.2% for those with regional disease, and 14.7% for patients with metastases. By Cox regression analysis, age and extent were significant prognostic factors of survival in patients with colon cancer; the risk was higher in patients with distant metastasis (hazard ratio [HR], 2.53; 95% confidence interval [CI], 1.17-5.45; p=0.01). In patients with rectal cancer, the risk was lower in males (HR, 0.66; CI, 0.45-0.98; p=0.04), but higher in patients with unknown tumor extent (HR, 3.70; 95% CI, 1.66-8.24; p=0.01).

**Conclusions:**

The five-year OS for 1994–2004 was 44.6% for patients with CRC. More so, five-year OS based on CRC stage was generally lower than the typically reported survival rates. The establishment of a national screening program and increased access to specialized medical faculties may be necessary to improve CRC survival in the KSA.

## Background

Cancer is a major public health problem in the Kingdom of Saudi Arabia (KSA) and many other countries. Worldwide, colorectal cancer (CRC) is the third most common cancer in men and the second in women. The highest incidence is in Australia, New Zealand and Western Europe, while the lowest is in Africa and South-Central Asia [1]. Both the incidence and mortality rates are lower in women than in men. The highest mortality in both genders is in Central and Eastern Europe, and the lowest is in Middle Africa [2].

The KSA is a low-risk country for CRC, but the incidence seems to be increasing [3]. Although CRC is less common in the KSA than in its counterpart Gulf Cooperation Council States and in the West, this disease was the second most common malignancy after breast cancer, ranking first among men and third among women between 1994 and 2004 [4]. In 2004, six hundred forty-seven new cases of CRC were diagnosed in the KSA. The median age at diagnosis was 60 years in men (range, 19–105 years) and 58 years in women (range 16–100 years) [4]. The age-standardized incidence rate in 2004 was 7.3 per 100,000 men and women based on cases diagnosed from 13 Saudi Cancer Registry (SCR) geographic sites. In the same year, the World Health Organization (WHO) reported that the age-standardized death rate (per 100,000 inhabitants) from CRC in the KSA was 8.3% [5].

In developing countries, the five-year survival rates range from 28% to 42% [6,7], compared with more than 60% in the US, Japan and Switzerland [8,9]. In the KSA, there are limited data on five-year survival rates for patients with CRC. Thus, the aim of this study was to review CRC data for the period 1994–2004 based on reports from the SCR in order to evaluate the prognostic factors for CRC survival in Saudi patients.

## Methods

This study was a retrospective censored overall survival (OS) analysis of CRC data for the period 1994–2004 obtained from the SCR. The SCR is a population-based registry that was established in 1992 under the jurisdiction of the Ministry of Health [5]. It endeavors to compile all cancer data from the Ministry of Health, private and governmental hospitals, clinics, and laboratories from all regions in the Kingdom.

Routine data were collected from all 13 administrative regions of the KSA by the SCR. The SCR collaborated with the National Information Center (NIC) of the Ministry of Interior through Al-Elm Information Security Company in order to calculate the five-year survival rates for Saudi patients with CRC. Because Al-Elm has access to huge government databases, they can verify the vital status of cancer patients through their Saudi national identity number.

A list of 15,484 patients (whose selection was based on the availability of their Saudi national identity number) was submitted to Al-Elm. Al-Elm analyzed only the data of patients whose ten-digit national identity number matched their complete four names in Arabic. Based on the above, the company provided the SCR with information on patient vital status and date of death (according to the Hijri calendar) if dead. The date of death was then converted to the equivalent Gregorian calendar date, and survival period was calculated from the date of diagnosis to the date of death or last follow up.

The Kaplan-Meier method was used to calculate the cumulative survival rate, and statistical significance was calculated with the log-rank test. The cumulative survival was then stratified by gender and by period (1994–1999 versus 2000–2004). Continuous variables were expressed as mean ± SD and categorical variables as frequency (percent). The clinico-pathological variables that might affect CRC survival were analyzed by Cox regression analysis. A p-value <0.05 was considered statistically significant.

The study was approved by the Biomedical Ethics Research Committee of King Abdulaziz University (reference number 816–12). To ensure confidentiality, the researchers signed a data use agreement.

## Results

Five thousand one hundred forty-one cases of cancer, including 549 of CRC were diagnosed between 1994 and 2004. Of the 549 cases of CRC, 363 were diagnosed in males (66.9%) and 186 in females (44.6%). As shown in Table 1, the mean age of the patients was 53.4 ± 14.7 years for patients with colon cancer, 53.6 ± 17.3 years for those with recto-sigmoid cancer, and 54.3 ± 15.2 years for those with rectal cancer. One hundred forty-two patients (25.9%) had advanced disease at diagnosis, and more than half of the patients (n=345; 62.8%) in the study population died during the study period.

**Table 1:** **Baseline characteristics of the patients**^**a**^

	**Cancer site**		
	**Colon(n, %)**	**Recto**-**sigmoid(n, %)**	**Rectum(n, %)**
**Number of patients**	257 (46.8)	66 (12.0)	226 (41.2)
**Age in years** (**Mean** ± **SD**)	53.4 ± 14.7	53.6 ± 17.3	54.3 ± 15.2
**Gender**			
Female	96 (51.6)	25 (13.4)	65 (34.9)
Male	161 (44.4)	41 (11.3)	161 (44.4)
**Grade**			
Grade I (Well diff)	26 (44.8)	5 (8.6)	27 (46.6)
Grade II (Mod diff)	157 (44.7)	48 (13.7)	146 (41.6)
Grade III (Poor diff)	25 (46.3)	3 (5.6)	26 (48.1)
Grade IV (Undiff anaplastic)	4 (66.7)	0 (0)	2 (33.3)
Unknown	45 (56.3)	10 (12.5)	25 (31.3)
**Extent**^b^			
Localized	48 (39.3)	14 (11.5)	60 (49.2)
Regional: Direct Ext	59 (54.6)	11 (10.2)	38 (35.2)
Regional: Lymph Node	12 (27.3)	9 (20.5)	23 (52.3)
Regional: Dir Ext and lymph node	64 (59.8)	9 (8.4)	34 (31.8)
Regional NOS	0 (0)	0 (0)	1 (100.0)
Distant Metastasis	63 (44.4)	21 (14.8)	58 (40.8)
Unknown	11 (44.0)	2 (8.0)	12 (48.0)
**Status**			
Dead	152 (44.1)	43 (12.5)	150 (43.5)
Alive	105 (51.5)	23 (11.3)	76 (37.3)
**Cause of death**			
Cancer	15 (62.5)	3 (12.5)	6 (25.0)
Unknown	137 (42.7)	40 (12.5)	144 (44.9)
Not applicable	105 (51.5)	23 (11.3)	76 (37.3)

Abbreviations: Diff, differentiation; Dir, direct; Ext, extent; Mod, moderate; NOS, not otherwise specified; Undiff, undifferentiated.

^a^Data are presented as frequency (percent) unless otherwise specified.

^b^p<0.05 for *χ*^2^ test. Except for extent, the characteristics of the patients did not differ significantly among different cancer patients.

The five-year OS for 1994–2004 was 44.6%. The five-year OS between 1994–1999 and 2000–2004 were similar (44.7% and 44.3%, respectively; p=0.7). There was a significant (p=0.003) discrepancy of 9.6% between the male five-year OS (41.0%) and the female five-year OS (50.6%) for the period 1994–2004.

**Figure 1:**
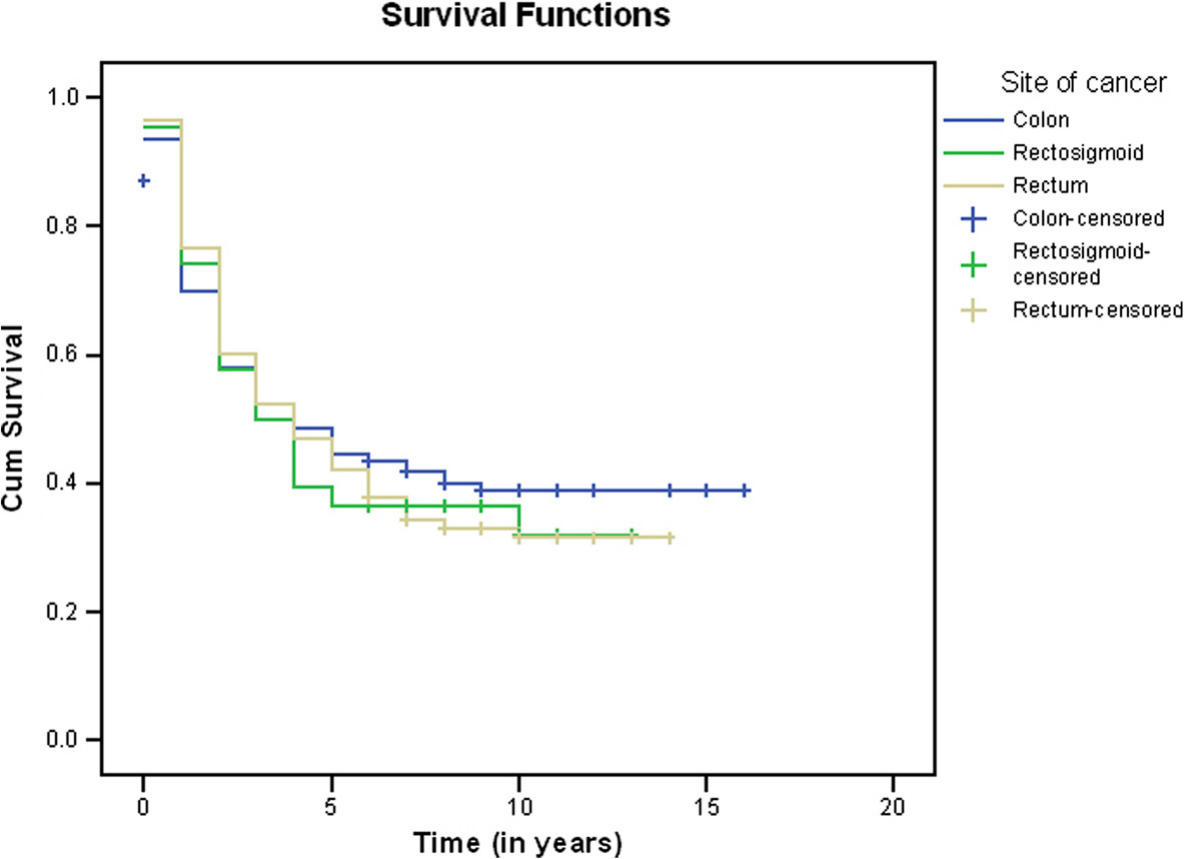
**Kaplan**-**Meier Curves for Colon**, **Recto-sigmoid, and Rectal Cancers.** Legend: The cumulative (Cum) survival for 1994–2004 for patients with colon, recto-sigmoid, and rectal cancers was 40.9, 34.8%, and 33.6%, respectively.

Based on cancer stage, the five-year OS rates for the Saudi population were 63.3% for patients who had localized disease, 50.2% for those who had regional disease, and 14.7% for patients with metastases. Overall survival rate was highest for colon cancer followed by recto-sigmoid and rectal cancers, respectively (Figure 1).

Results of Cox regression analysis are shown in Table 2. Age and distant metastasis were significant prognostic factors of survival in patients with colon cancer. The risk of death was higher in patients with distant metastasis (HR, 2.53; 95% CI, 1.17-5.45; p=0.01). The risk was also higher in patients >65 years (hazard ratio [HR], 0.65; 95% confidence interval [CI], 0.43-0.97; p=0.03) than in those aged 45–65 years (HR, 0.52; 95% CI, 0.33-0.81; p=0.01). Gender and grade were not significant risk factors.

**Table 2:** **Results of specific Cox regression models**^**a**^

**Variables**	**HR**	**95%CI**	**P**-**value**
**Colon**			
**Age**			
<45 years (Ref)	-	-	-
45-65 years	0.52	0.33 - 0.81	0.01
>65 years	0.65	0.43 - 0.97	0.03
**Gender**			
Female (Ref)	-	-	-
Male	0.74	0.52 - 1.05	0.10
**Grade**			
Grade I (Well diff) (Ref)	-	-	-
Grade II (Mod diff)	0.72	0.39 - 1.34	0.30
Grade III (Poor diff)	0.66	0.43 - 1.01	0.06
Grade IV (Undiff Anaplastic)	1.12	0.63 - 2.01	0.68
Unknown	0.55	0.13 - 2.37	0.42
**Extension**			
Localized (Ref)	-	-	-
Regional: Direct Ext	0.45	0.18 - 1.11	0.08
Regional: Lymph Node	0.65	0.28 - 1.51	0.32
Regional: Dir Ext and Lymph Node	0.81	0.25 - 2.58	0.72
Regional NOS	1.44	0.66 - 3.15	0.35
Distant Metastasis	2.53	1.17 - 5.45	0.01
**Recto**-**sigmoid**			
**Age**			
<45 years (Ref)	-	-	-
45-65 years	0.53	0.19 - 1.46	0.22
>65 years	0.61	0.26 - 1.41	0.25
**Gender**			
Female (Ref)	-	-	-
Male	1.07	0.45 - 2.55	0.86
**Grade**			
Grade I (Well diff) (Ref)	-	-	-
Grade II (Mod diff)	0.85	0.19 - 3.73	0.83
Grade III (Poor diff)	0.55	0.21 - 1.48	0.24
Grade IV (Undiff Anaplastic)	0.88	0.20 - 3.84	0.86
**Extent**			
Localized (Ref)	-	-	-
Regional: Direct Ext	0.74	0.07 - 7.41	0.79
Regional: Lymph Node	1.83	0.17 - 18.95	0.61
Regional: Dir Ext and Lymph Node	2.12	0.21 - 20.94	0.52
Regional NOS	2.13	0.21 - 21.84	0.52
Distant Metastasis	5.74	0.67 - 49.01	0.11
**Rectum**			
**Age**			
<45 years (Ref)	-	-	-
45-65 years	0.66	0.41 - 1.05	0.08
>65 years	0.72	0.48 - 1.08	0.11
**Gender**			
Female (Ref)	-	-	-
Male	0.66	0.45 - 0.98	0.04
**Grade**			
Grade I (Well diff) (Ref)	-	-	-
Grade II (Mod diff)	0.63	0.32 - 1.21	0.16
Grade III (Poor diff)	0.78	0.48 - 1.29	0.34
Grade IV (Undiff Anaplastic)	1.52	0.80 - 2.87	0.19
Unknown	0.75	0.09 - 6.04	0.79
**Extension**			
Localized (Ref)	-	-	-
Regional: Direct Ext	1.11	0.49 - 2.51	0.79
Regional: Lymph Node	1.01	0.42 - 2.45	0.97
Regional: Dir Ext and Lymph Node	0.86	0.33 - 2.24	0.75
Regional NOS	1.48	0.63 - 3.47	0.36
Distant Metastasis	3.92	0.46 - 33.25	0.21
Unknown	3.70	1.66 - 8.24	0.01

Abbreviations: CI, confidence interval; Diff, differentiation; Dir, direct; Ext, extent; HR, hazard ratio; Mod, moderate; NOS, not otherwise specified; Ref, reference, Undiff, undifferentiated.

In patients with rectal cancer, the risk was lower in males (HR, 0.66; CI, 0.45-0.98; p=0.04), but higher in patients with unknown tumor extent (HR, 3.70; 95% CI, 1.66-8.24; p=0.01). Age and tumor grade were not significant prognostic factors in these patients (Table 2).

Age, gender, tumor grade and extent were not significant factors of survival in patients with recto-sigmoid cancer (Table 2).

## Discussion

The incidence of CRC among the Saudi population has increased continuously over the last decade as in many developing countries where the overall risk was previously low [6]. In contrast, the incidence of CRC is declining in the United States and in many developed countries worldwide [10,11]. The geographic differences in CRC incidence, as seen in studies of migrants moving from low- to high-risk areas [7,8], are probably explained by environmental factors and changing dietary patterns. Major risk factors that have been identified include physical inactivity, high body mass index, and central adiposity [7,12,13]. Other risk factors include a diet poor in fiber and rich in meat and fats [7,9], cigarette smoking, and genetic predisposition [14,15]. Similarly, family history and a higher consumption of meat and fats from animal sources were associated with a greater risk of developing CRC in studies conducted in the KSA [16-18].

CRC survival largely depends upon the stage of the disease at diagnosis. Five-year survival rates for CRC typically range from 90% for localized cancers, 70% for regional cancers, to 10% for distant metastatic cancers [19,20]. Over the last five decades, survival for CRC at all stages has increased considerably [19]. The relative improvement in the five-year survival rates during this period is reportedly better in countries with high life-expectancy and improved access to specialized health care services. In this study, OS rates based on CRC stage are generally lower than the typically reported survival rates. The five-year OS for 1994–2004 was 63.3% for patients with localized disease, 50.2% for those with regional disease, and 14.7% for patients with metastatic disease. Lack of cancer-preventing and screening programs, accessibility to specialized centers, and efficient diagnostic techniques to improve diagnosis, prognosis, and hence survival [21] probably explain the lower survival rates in the KSA. These could also explain the lack of improvement in the five-year OS between the periods 1994–1999 and 2000–2004.

There was a significant discrepancy of 9.6% between the male five-year OS (41.0%) and the female five-year OS (50.6%). In addition, gender was a significant prognostic factor of survival in patients with rectal cancer; however, there is no clear explanation for these findings. According to the observations of some authors, the lifetime risk of dying from CRC is similar in both genders because the life expectancy is on average higher in women than men [21]. More recently, patient gender has been extensively evaluated and most studies showed that it was of no significance in predicting survival independently of other factors [22-24].

As with most cancers, relative survival for CRC is higher in patients younger than 70 years, even after the higher background mortality in older persons is taken into consideration [25]. In this study, age and tumor extent were significant prognostic factors for survival in patients with colon cancer. Several studies have demonstrated that old age and advanced disease stage were prognostic factors associated with poor prognosis in patients with CRC [26-29]. However, old age was reported to be an independent prognostic factor, and because it may be associated with cardiovascular diseases or other illnesses [30], overall survival tends to be poor and not cancer-specific in older patients with CRC.

In several studies conducted abroad, other clinico-pathological prognostic factors have been proposed, including tumor location [31,32] and differentiation [23,31]. Unfortunately, there are no data that evaluate the prognostic factors for CRC survival in Saudi patients. In this study, patients with colon cancer had a better survival than those with rectal cancer similar to the findings of other authors [33]. Nevertheless, it seems that among all the pathological factors that have been explored, those that are related to early diagnosis of CRC cases are the most important [33]. Thus, screening and early detection of CRC should be a priority in Saudi health care programs.

This study has some limitations. Given that the SCR was only recently established, mortality data were not available for patients with CRC in the KSA. Furthermore, five-year relative OS could not be established as all deaths were registered based on information from Al-Elm, which could only verify the vital status of cancer patients through their Saudi national identity number.

## Conclusions

The five-year OS for 1994–2004 was 44.6% for patients with CRC. More so, five-year OS based on CRC stage is generally lower than the typically reported survival rates. This raises concerns about CRC diagnosis and care in the KSA. The establishment of a national screening program and increased access to specialized medical faculties may be necessary to improve CRC survival in the KSA. Further exploration may be necessary to explain the 9.6% difference in five-year OS between males and females.

## Abbreviations

CRC: Colorectal cancer; KSA: Kingdom of Saudi Arabia; OS: Overall survival; SCR: Saudi cancer registry; US: United States.

## Competing interests

The authors declare that they have no competing interests.

## Authors’ contributions

MSA designed and wrote the proposal for the study and submitted it to the research committee of King Abdulaziz University. SY and AH helped with the statistical analysis and literature review. All authors contributed to writing the manuscript. All authors read and approved the final manuscript.

## Pre-publication history

The pre-publication history for this paper can be accessed here:

http://www.biomedcentral.com/1471-2458/13/73/prepub
